# A prospective comparison of fiberoptic endobronchial needle aspiration, bronchial brushing, and forceps biopsy for the diagnoses of canine exophytic tracheal and endobronchial masses, and submucosal infiltrations

**DOI:** 10.1111/jsap.13891

**Published:** 2025-06-04

**Authors:** D. De Lorenzi, G. Maggi, D. Bertoncello, E. Bottero, M. C. Marchesi

**Affiliations:** ^1^ San Marco Veterinary Clinic and Laboratory Padova Italy; ^2^ Department of Veterinary Medicine University of Perugia Perugia Italy; ^3^ Department of Interventional Pulmonology and Ear‐Nose‐Throat Medicine and Surgery Anicura Veterinary Hospital I Portoni Rossi Bologna Italy; ^4^ Veterinary Clinic “Città di Torino” Torino Italy

## Abstract

**Objectives:**

To compare the diagnostic yield of endobronchial Wang™ needle aspiration to that of bronchial brushing and forceps biopsy for canine tracheal and endobronchial masses and submucosal infiltrations examined by fiberoptic bronchoscopy.

**Materials and Methods:**

Flexible fiberoptic bronchoscope‐guided bronchial brushing, forceps biopsy, and endobronchial Wang™ needle aspiration were performed consecutively in dogs with exophytic airway masses or submucosal infiltrations. The diagnostic performances of the three techniques were compared to surgical or necropsy histopathology, as the gold standard. We determined the diagnostic sensitivity, specificity, positive predictive value, negative predictive value, accuracy, and 95% confidence interval of each technique.

**Results:**

Twenty‐one dogs were included. Endobronchial Wang™ needle aspiration accurately identified malignancy in 90.48% of cases, forceps biopsy in 80.95%, and bronchial brushing in 52.38%. Of the 21 cases, agreement in the final morphological tumour type was obtained in 19 (90%), 15 (71%), and 8 (38%) using endobronchial Wang™ needle aspiration, forceps biopsy, and bronchial brushing, respectively. Endobronchial Wang™ needle aspiration had the highest sensitivity and accuracy (94% and 90%, respectively; 95% CI: +0.99/−0.89) when used both alone and in combination with the other techniques.

**Clinical Significance:**

Endobronchial Wang™ needle aspiration alone or in combination with other techniques may be promising for obtaining the highest diagnostic yield for canine tracheal or bronchial mucosal abnormalities.

## INTRODUCTION

Primary lung and bronchial tumours are uncommon in companion animals, with an occurrence rate of approximately 1%, in contrast to the higher incidence observed in humans (Moulton et al., 1981; Withrow, 2007; Zhang et al., 2023). Mucosal changes caused by primary or metastatic lung tumours are infrequently observed in dogs because these tumours typically develop in the peripheral regions. Exophytic endobronchial masses or mucosal invasions, which are typical of human lung cancer, are uncommon in dogs (De Lorenzi, 2012; Moulton et al., 1981; Zhang et al., 2019). Exophytic endobronchial masses in dogs arise from neoplastic or inflammatory causes (Brownlie, 1990; De Lorenzi & Solano‐Gallego, 2009; Hill et al., 2008). Common non‐malignant causes of mucosal airway abnormalities include inflammatory conditions such as chronic bronchitis and eosinophilic bronchopneumopathy (Clercx et al., 2000; Zhu et al., 2015). Flexible bronchoscopy is the most useful noninvasive technique for investigating endobronchial abnormalities and is considered essential for definitively diagnosing lung tumours (Kalvapudi et al., 2024). Although the bronchial epithelium generally responds to irritation in limited ways, visible changes may not be pathognomonic for a specific disease (Zhu et al., 2015). Therefore, airway samples are necessary to establish a diagnosis.

Historically, clinicians have used a combination of established conventional diagnostic techniques such as bronchial forceps biopsy (FB), bronchial brushing (BB), and bronchial washing (BW) (Padrid, 2010). This group has since been expanded to include endobronchial needle aspiration (EBNA) owing to the increasing recognition of its diagnostic utility in endobronchial lung cancer (Dobler & Crawford, 2009; Mohamed et al., 2013). EBNA is an important bronchoscopic sampling technique that is commonly used in human medicine for malignancy diagnosis and staging (Liu et al., 2015; Lundgren et al., 1983). It is routinely performed for sampling submucosal endobronchial lesions (e.g., erythema, mucosa thickening, loss of bronchial markings) or masses that compress the bronchial lumen extrinsically, and is occasionally used for sampling exophytic endobronchial tumors (Horsley et al., 1984; Lundgren et al., 1983). The routine use of EBNA is associated with minimal patient risk, although potential complications include bleeding and iatrogenic pneumothorax (Padrid, 2010). Although the use of EBNA with the Wang™ needle has been reported in veterinary literature, its routine application has not yet been established (De Lorenzi, 2012; Dear & Johnson, 2013; Falcioni et al., 2022).

The PubMed and Google Scholar databases were searched using the following keywords: airway, sample collection, and small animals on 09/02/2025, and four textbooks were consulted (McCarthy T.C. Veterinary Endoscopy for the Small Animal Practitioner; Tams T.R. & Rawlings C.A. Small Animals Endoscopy; De Lorenzi D. Diseases of the Respiratory System of the Dog and Cat; and Enrico B. Interventional Endoscopy in the Dog and Cat).

The aim of this study was to compare the diagnostic yield and complications of EBNA to those of BB and FB in dogs with exophytic masses (EM) or submucosal infiltrations (SI) examined by fiberoptic bronchoscopy. We specifically focused on (1) distinguishing between benign and malignant lesions and (2) establishing a correct cytological diagnosis.

## MATERIALS AND METHODS

### Animals

All dogs with a radiographic and/or tomographic diagnosis of a tracheal or bronchopulmonary mass between January 2023 and December 2023 at Anicura Hospital I Portoni Rossi of Zola Predosa (Bologna, Italy) were eligible for the study. Included dogs had visible endotracheal or endobronchial abnormalities during bronchoscopy. These abnormalities included EM or SI, the latter defined as thickening or loss of mucosal markings with tracheal or bronchial narrowing. Dogs with normal mucosa but apparent airway narrowing due to extrinsic or extramural compression were not included. Dogs were included if they underwent EBNA, BB, and FB, and if a definitive histopathological diagnosis from a surgical biopsy or necropsy had been reached. Age, breed, sex, neuter status, and endoscopic findings were recorded before study inclusion. Informed consent was obtained from all dog owners, who were briefed on the three sampling techniques involved.

### Intervention

All bronchoscopies were performed by experienced operators (DDL and DB) and all cytological samples were interpreted by a board‐certified clinical pathologist (DDL). Bronchoscopic examination in all dogs was performed in a standardised manner using a flexible fiberoptic bronchoscope (Fibroscope 60003VB, KARL STORZ, Tuttlingen, Germany). This was conducted under general anaesthesia comprising premedication with 2 μg/kg of dexmedetomidine and 0.2 mg/kg of methadone intramuscularly and induction with 4 mg/kg of propofol administered intravenously. A 5‐minute preoxygenation period was used. A stable plane of anaesthesia was maintained with a constant‐rate infusion of propofol at 0.1 to 0.4 mg/kg/minute or intermittent boluses. Oxygen was delivered through the working channel of the bronchoscope or via jet ventilation. Electrocardiography, blood pressure, and pulse oximetry were constantly monitored. During recovery, supplemental oxygen was administered as required. The sequence of sampling was as follows: BB, EBNA, and FB.

BB cytology was performed using a sheathed disposable cytology brush (code no. 9939; Aorta S.r.l., Milano, Italy). After each brushing, the brush was sheathed and withdrawn from the bronchoscope. The brush was smeared by gentle rolling onto a glass slide. This procedure was repeated to obtain two separate brush specimens from each lesion in order to minimize the number of cases in which brush samples were inadequate for cytological interpretation. The slides were air‐dried and stained with May‐Grünwald‐Giemsa (MGG) using an automatic slide stainer (Aerospray Slide Stainer 7000; WESCOR, Logan, UT, USA).

EBNA was performed using 22‐gauge transbronchial aspiration needles (Wang™ Transbronchial Cytology Needle; CONMED Corporation, Utica, NY, USA). The Wang™ needle comprises an outer flexible plastic catheter, a distal retractable sharp bevelled needle (15 mm in length), a middle flexible catheter, a stylet, a proximal device for needle control, and a side port for suction application. The outer plastic catheter has a metallic hub at its distal end to protect the working channel of the endoscope from needle‐induced perforations. The middle flexible catheter transmits negative pressure from the proximal end to the distal needle, while the stylet provides rigidity for successful insertion through hard masses and prevents clogging of bronchial epithelial cells in submucosal masses. Owing to the nature of the procedure, both catheters have sufficient flexibility to be manoeuvred into peripheral locations, while remaining stiff enough to exert the force necessary to penetrate both soft and hard masses. To obtain a specimen, the Wang™ needle was inserted into the working channel of the fibroscope. To prevent needle damage to the working channel of the fibroscope, the fibroscope was kept as straight as possible with the distal tip in a neutral position during catheter insertion. The bevelled end of the needle was secured within the metal hub during passage through the working channel, and the needle was advanced and locked in place only after the metallic hub became visible beyond the tip of the fibroscope. Once the needle tip penetrated the mass, the internal sheath was removed, and rapid negative pressure was applied by multiple aspirations with a 20‐mL syringe. After the needle was moved back and forth inside the mass, it was retrieved and the internal sheath was removed. Positive pressure was then applied using an air‐filled 20‐mL syringe to expel the aspirated material onto a glass slide, which was then smeared against another glass slide using the squash preparation technique to yield cytological smears on two glass slides, as previously described (De Lorenzi et al., 2008). Slides were air‐dried and stained with MGG using the automatic slide stainer (Aerospray Slide Stainer 7000). Two aspirated samples were collected from each lesion.

Endobronchial FB was performed using flexible biopsy forceps (MicroTech BF‐18‐12AC‐1, Nanjing, China) introduced into the working channel of the bronchoscope. Three FBs were obtained from each lesion unless the collection of samples was limited by complications such as bleeding. All histological specimens were placed on biopsy sponges, fixed in 10% neutral‐buffered formalin, processed, and embedded in paraffin wax. Four‐millimetre sections were stained with haematoxylin and eosin, and then evaluated by a pathologist who was blinded to the cytological diagnosis.

Cytopathological specimens were classified into six groups based on the cytomorphological criteria described in the veterinary literature (Hecht, 2008; McMillian et al., 1988; Wood et al., 1998): (1) carcinoma, (2) neuroendocrine tumour, (3) sarcoma, (4) round cell tumour, (5) other neoplastic lesions, and (6) non‐neoplastic lesions.

### Outcome assessment

We compared the sensitivity, specificity, positive predictive value (PPV), negative predictive value (NPV), accuracy, and 95% confidence interval (CI) of EBNA alone to correctly distinguish between benign and malignant growth, and establish a correct cytological diagnosis based on surgical or necropsy histopathology, as the gold standard. These results were further compared to those obtained from BB and FB sampling. We also compared the sensitivity, specificity, PPV, NPV, accuracy, and 95% CI of all possible sampling combinations (EBNA and BB; EBNA and FB; BB and FB; and EBNA, FB, and BB) for the correct distinction between benign and malignant growths.

### Statistical methods

Only descriptive statistics were provided. The results were expressed as mean and standard deviation (SD) for continuous variables, and number (%) for qualitative and semi‐quantitative variables.

## RESULTS

Of the 26 eligible dogs, 21 were enrolled in the study. Three dogs were excluded due to concerns regarding possible bleeding complications after EBNA that precluded subsequent FB. Two dogs were excluded due to the lack of a definitive histopathological diagnosis from a surgical specimen or necropsy. The most common breeds included were mixed‐breed (*n* = 8, 38%), English Setter (*n* = 2, 10%), and Labrador retriever (*n* = 2, 10%). Several other breeds were also represented including Boxer, Dalmatian, Dobermann, German shepherd, Golden retriever, Fox terrier, Schnauzer, Springer spaniel, and Rottweiler (one case of each). There were four (19%) spayed females, three (14%) intact females, 12 (57%) intact males, and two (10%) neutered males. The mean age was 10.7 years (SD = 3.75 years; range: 1 to 16 years).

The endotracheal or endobronchial endoscopic abnormalities detected were EM in 14 of 21 cases (67%) and SI in seven (33%). The distribution of endoscopic findings was focal in all cases, except for one, which had multifocal localization of bronchial SIs. In six dogs (29%), the abnormalities were localized in the trachea; of these, four were EM and two were SI. The remaining 15 endoscopic lesions (71%) were located in the segmental or subsegmental bronchi. No complications were encountered when obtaining the BB and FB samples. While no major complications were reported during the EBNA procedure, two dogs experienced a minor complication, primarily moderate bleeding, preventing collection of FB, leading to exclusion from the study.

A definitive histopathological diagnosis was established for all dogs based on necropsy or surgical specimens. Malignancy was found in 17 cases (81%), whereas non‐malignant lesions were identified in four (19%). The most common tumour type was carcinoma, followed by sarcoma, carcinoid, melanoma, and mast cell tumours. The most common non‐neoplastic lesions were abscesses, followed by granulomas and chondromas. The results of the BB, EBNA, FB, and histopathological definitive diagnoses are shown in Table 1. EBNA yielded the highest number of positive results for malignancies, followed by FB and BB. Compared to the histopathological diagnosis, EBNA correctly diagnosed the benign or malignant nature of the lesion in 19 of 21 cases (90%), FB in 17 (81%), and BB in only 11 (52%). Similar results were obtained for diagnostic accuracy. Endobronchial needle aspiration agreed with the final morphological tumour type or diagnosis in 19 of 21 cases (90%), FB in 15 (71%), and BB in only eight (38%). The results of sensitivity, specificity, accuracy, PPV, NPV, and 95% CI for the various techniques and combinations are given in Table 2. The technique with the highest sensitivity for detecting malignancy was EBNA, both alone and in combination with the other techniques. Diagnostic accuracy was the highest when the techniques were combined, particularly when EBNA was used in conjunction with FB or BB.

**Table 1:** BB‐, EBNA‐, and FB‐derived cytologic or histologic diagnoses, and the definitive diagnoses of the 21 dogs included in the study

	Bronchial brushing (BB) (*n* = 21)	Endobronchial Wang™ needle aspiration (EBNA) (*n* = 21)	Forceps biopsy (FB) (*n* = 21)	Histologic definitive diagnosis (*n* = 21)
Malignancy	7 (33%)	17 (81%)	13 (62%)	17 (81%)
1. Carcinoma	5 (24%)	9 (43%)	8 (38%)	10 (48%)
2. Sarcoma	1 (5%)	4 (19%)	2 (10%)	3 (14%)
3. Neuroendocrine tumour	–	2 (10%)	–	–
Carcinoid	–	–	–	2 (10%)
4. Round cell tumour				
Mast cell tumours	1 (5%)	1 (5%)	1 (5%)	1 (5%)
5. Other neoplastic lesions				
Melanoma	–	1 (5%)	1 (5%)	1 (5%)
Unspecified malignant tumour	–	–	1 (5%)	–
Non‐neoplastic lesions	14 (67%)	4 (19%)	8 (38%)	4 (19%)
Normal mucosa	1 (5%)	–	–	–
Necrosis and/or phlogosis and/or dysplasia	13 (62%)	4 (19%)	7 (33%)	–
Abscess	–	–	–	2 (10%)
Granuloma	–	–	–	1 (5%)
Chondroma	–	–	1 (5%)	1 (5%)

**Table 2:** Sensitivity, specificity, accuracy, positive predictive value, and negative predictive value of the three techniques (BB, ENBA, and FB) and their combinations

	Sens.	Spec.	Acc.	PPV	NPV	95% CI
BB	29%	100%	42%	100%	25%	±1
ENBA	94%	75%	90%	94%	75%	+0.99/−0.89
FB	76%	100%	80%	100%	5%	±1
BB + ENBA	100%	75%	95%	94%	100%	+0.99/−0.89
BB + FB	82%	100%	85%	100%	57%	±1
FB + ENBA	100%	66%	95%	94%	100%	+0.99/−0.89
ENBA + BB + FB	82%	100%	85%	100%	57%	±1

Acc., Accuracy; BB, Bronchial brushing; ENBA, Endobronchial Wang™ needle biopsy aspiration; FB, Forceps biopsy; NPV, Negative predictive value; PPV, Positive predictive value; Sens., Sensitivity; Spec., Specificity; 95% CI, 95% Confidence interval

## DISCUSSION

EBNA is not routinely used in veterinary medicine. To the best of our knowledge and review of the literature, this is the first study to explore the use of EBNA for the diagnosis of airway diseases in small animals. EBNA detected lower airway malignancies in many cases with histopathological neoplasms identified during necropsy or surgery. It also had the highest sensitivity for detecting malignancy compared to BB and FB. Its diagnostic power was enhanced when used in conjunction with BB and FB. Similar results were observed for non‐neoplastic lesions.

BB is a complementary airway diagnostic method that allows the direct cytological evaluation of visible endobronchial lesions. The use of BB for the detection of bronchial inflammatory diseases has been widely validated in veterinary medicine; however, data regarding its use for malignancy detection are limited (Zhu et al., 2015). In a study by Zhu et al. (2015), comparing BB and bronchoalveolar lavage (BAL) in dogs with chronic cough, BB failed to detect malignancy in a case of carcinoma, instead revealing neutrophilic inflammation along with hyperplasia and dysplasia (Zhu et al., 2015). Our results are consistent with the data from this previous study. In 10 of 17 cases, BB identified necrosis, inflammation, and/or dysplasia in the presence of neoplasia. In human medicine, BB has a sensitivity of up to 71% for a malignancy diagnosis, whereas in veterinary medicine, its sensitivity for detecting neoplasms is unreported (Zhu et al., 2015). In this study, BB had a sensitivity, specificity, and accuracy of 29%, 100%, and 42%, respectively. It had the lowest sensitivity and accuracy among the techniques investigated. However, its diagnostic capacity improved when used in combination with FB and EBNA.

Although endobronchial biopsy is less common in veterinary medicine than in human medicine, it is still a valuable diagnostic tool. In human medicine, FB has a sensitivity of up to 74% for cancer diagnosis, whereas in veterinary medicine such data are lacking (Govert et al., 1999). In this study, FB had a sensitivity, specificity, and accuracy of 76%, 100%, and 80%, respectively. According to previous human studies, the sensitivity of FB is higher when this sampling technique is used in combination with other methods, particularly EBNA. EBNA has been shown to be more sensitive and accurate than FB for both malignant and nonneoplastic lesions. This could be attributed to the size of the samples obtained using FB. Samples obtained using endoscopic forceps are typically small (<2 mm), and interpretation may be difficult (McKiernan, 2005; Padrid, 2010). The mucosa retrieved from the FB is usually 1 to 1.5 mm in size and is frequently crushed. The presence of crush artefacts and their small size makes histological interpretation of the sample difficult (Padrid, 2010). Additionally, the samples obtained by FB involve more superficial portions of the lesions, with a higher possibility of sampling the inflammatory or necrotic perilesional areas. In human medicine, some types of tumours are characterised by a higher cellular volume in the central portion, which in some cases leads to a better diagnostic yield with EBNA than with FB (Mohamed et al., 2013). These factors can also influence the diagnostic sensitivity of the technique. When a biopsy is performed, multiple specimens are obtained, if possible, to provide the pathologist with a greater chance of making a correct diagnosis. In this study, two samples were obtained from each dog by FB. Although a direct comparison of sensitivity between one or two samples was not conducted, EBNA consistently demonstrated higher sensitivity and accuracy.

There is a lack of information on the diagnostic yield of EBNA in veterinary medicine because it is not routinely performed. In this study, EBNA had a sensitivity, specificity, and accuracy of 94%, 75%, and 90%, respectively. It had the greatest sensitivity and accuracy compared to the other techniques assessed. This appeared to be true regardless of whether its sensitivity for tumors only or for all lesions was considered. In only one case, EBNA was used to collect necrotic tissue, and the definitive diagnosis was carcinoma. We suspect that EBNA is more effective than BB or FB at diagnosing airway abnormalities. This is owing to the ability of the needle to easily penetrate the mucosa and submucosa of a lesion, thereby avoiding the sampling of the inflammation and necrosis potentially surrounding the neoplastic tissue. One potential disadvantage of EBNA in lung tumor diagnosis is its potential for misclassification of cell types. In one case, sarcoma was identified when the definitive diagnosis was chondroma. However, none of the previous techniques had a greater accuracy than EBNA. Furthermore, in accordance with previous human studies, the use of a combination of EBNA, FB, and BB is highly sensitive and accurate for the diagnosis of airway diseases and cancer (Govert et al., 1999). The combination of these methods could enhance the diagnostic sensitivity by increasing the number of samples, thereby increasing the likelihood of obtaining diagnostic‐quality samples and reducing the risk of false‐negative results.

In our series of dogs, EBNA appeared to provide cytological samples of diagnostic quality, characterized by the absence of artefacts and sufficient cellularity for accurate analysis. This can be considered as an additional advantage of EBNA over FB because it allows the acquisition of a diagnostic sample through a single sampling. Moderate bleeding, observed as a minor complication during EBNA, prevented the subsequent performance of the FB procedure. However, this complication did not result in any clinical issues for the affected dogs. EBNA is occasionally associated with persistent bleeding and pneumothorax (Padrid, 2010). For these reasons, a flexible bronchoscope should be maintained at the sampled sites to monitor bleeding, and the vital parameters of the animals should be monitored to rapidly identify signs of iatrogenic pneumothorax.

Our study has some limitations. The main limitation was the small number of animals included owing to the low incidence of this type of lesion in dogs. This may explain the wide CI observed. A small cohort inherently reduces statistical power and increases variability, which can affect the reliability of the calculated metrics such as sensitivity, specificity, and accuracy. Consequently, although promising, our findings should be interpreted with caution as they may not be fully representative of the broader population. Another limitation was the absence of a comparison between the endoscopy‐guided techniques analysed (BB, FB, and EBNA) and the methods commonly used to evaluate pulmonary lesions in veterinary medicine, such as transthoracic fine‐needle aspiration (FNA) and BAL. The intraluminal localization of the airway lesions described in our study rendered the use of FNA (which is suitable for sampling pulmonary areas adjacent to the thoracic wall) less appropriate. Similarly, BAL is more suitable for collecting cells originating from the alveoli (De Lorenzi, 2012). Another consideration pertains to the routine use of BAL in veterinary medicine. BAL is a valuable diagnostic tool for various respiratory tract diseases. Unlike in human medicine, where this sampling method is primarily employed for identifying infections through collected fluid culture, in small animals, BAL is typically used for cytological analysis (De Lorenzi, 2012). This allows the identification of inflammatory cell populations and, less commonly, the detection of bacterial, fungal, or parasitic infections (De Lorenzi, 2012; Siler et al., 2023). A few studies have reported the use of BAL for detecting airway neoplasms, revealing that it is specific, but insensitive for the diagnosis of pulmonary neoplasia in small animals (Siler et al., 2023).

Although EBNA requires specialised instruments and the expertise of a skilled practitioner, it has the highest diagnostic sensitivity and accuracy compared with FB and BB. Its diagnostic efficacy was further improved when used in combination with the other two sampling techniques. Moreover, EBNA can be performed with minimal risk to animals. Furthermore, no major complications occurred when using EBNA in combination with other complementary sampling methods. EBNA resulted in bleeding in only two dogs, which prevented FB. Although this technique proved to be safe, sensitive, and accurate in our series of dogs, further studies with larger sample sizes should be performed to validate our findings. Future studies should focus on comparing EBNA with other lung tumour cell sampling techniques such as transthoracic FNA and BAL.

## Author contributions

**D. De Lorenzi:** Conceptualization (equal); data curation (lead); formal analysis (equal); investigation (lead); project administration (lead); validation (lead); visualisation (lead); writing – original draft (lead); and writing – review and editing (equal). **G. Maggi:** Conceptualization (equal); data curation (lead); formal analysis (equal); investigation (supporting); project administration (lead); validation (lead); visualisation (lead); writing – original draft (lead); and writing – review and editing (equal). **D. Bertoncello:** Data curation (supporting); investigation (lead); visualisation (supporting); and writing – review and editing (supporting). **E. Bottero:** Conceptualization (supporting); data curation (supporting); investigation (supporting); visualisation (supporting); and writing – review and editing (supporting). **M. C. Marchesi:** Conceptualization (equal); data curation (supporting); formal analysis (lead); investigation (supporting); project administration (equal); supervision (lead); validation (equal); visualisation (equal); and writing – review and editing (equal).

## Funding information

This research did not receive any specific grants from funding agencies in the public, commercial, or not‐for‐profit sectors.

## Conflict of interest

No conflicts of interest have been declared.

## Data Availability

The data that support the findings of this study are available from the corresponding author upon reasonable request.
